# Prognostic Significance of Complete Blood Count-Derived Inflammatory Biomarkers in Patients with Small Cell Neuroendocrine Carcinoma of the Cervix

**DOI:** 10.3390/curroncol32120654

**Published:** 2025-11-21

**Authors:** Mingxuan Zhu, Jing Liu, Yuqin Wang, Huaiwu Lu, Qin Xu

**Affiliations:** 1 Departments of Gynecology, Clinical Oncology School of Fujian Medical University, Fujian Cancer Hospital (Fujian Branch of Fudan University Shanghai Cancer Center), Fuzhou 350014, China; 2 Department of Gynecological Oncology, Sun Yat-Sen Memorial Hospital, Sun Yat-Sen University, Guangzhou 510123, China; 3 Guangdong Provincial Key Laboratory of Malignant Tumor Epigenetics and Gene Regulation, Sun Yat-Sen Memorial Hospital, Sun Yat-Sen University, Guangzhou 510123, China

**Keywords:** small cell neuroendocrine carcinoma of the cervix, inflammatory biomarkers, monocyte-to-lymphocyte ratio, prognostic model, nomogram

## Abstract

Background: Small cell neuroendocrine carcinoma of the cervix (SCNEC) is a rare and highly aggressive malignancy with limited prognostic biomarkers available for clinical use. Inflammatory markers derived from complete blood count (CBC) have been shown to reflect the systemic immune response and tumor progression in various cancers, but their prognostic value in SCNEC remains unclear. Methods: We retrospectively analyzed clinical data from patients diagnosed with SCNEC between 2004 and 2024 across two centers. Internal validation was performed by dividing patients into training and test cohorts. Cox regression analyses and Kaplan–Meier survival analyses were used to evaluate prognostic factors and treatment outcomes. Inverse probability of treatment weighting (IPTW) was applied to reduce baseline imbalances. Patients were randomly divided into training and test cohorts. A nomogram was constructed to predict 3-year and 5-year progression-free survival (PFS) with performance evaluated using receiver operating characteristic (ROC) curves, calibration plots, and decision curve analysis (DCA). Results: 196 participants were included in the study. Age, FIGO 2018 stage, surgery, neutrophil-to-lymphocyte ratio (NLR), and monocyte-to-lymphocyte ratio (MLR) were independently associated with PFS. High MLR (>0.19) was significantly linked to shorter PFS. After IPTW adjustment, the protective effect of low MLR on PFS remained significant (*p* = 0.029). The constructed nomogram demonstrated excellent predictive performance, with area under the curve (AUC) values of 0.799 and 0.787 for 3-year and 5-year PFS in the training cohort, and 0.802 for endpoints in the test cohort. Conclusions: MLR was identified as an independent prognostic biomarker for PFS in SCNEC, with potential value in risk stratification and personalized treatment strategies. Additionally, we developed a reliable nomogram that accurately predicts 3-year and 5-year PFS, serving as a practical tool for individualized prognosis and clinical decision-making.

## Simple Summary

Small cell neuroendocrine carcinoma of the cervix (SCNEC) is an uncommon but highly aggressive cancer with limited prognostic indicators to guide clinical decisions. Routine blood tests contain valuable information that may reflect the body’s immune and inflammatory response to cancer. In this study, we analyzed blood-derived inflammatory markers in patients with SCNEC and identified the monocyte-to-lymphocyte ratio (MLR) as a promising indicator of disease progression. We further developed a prediction tool, known as a nomogram, that estimates each patient’s likelihood of remaining progression-free at 3 and 5 years. This model demonstrated strong accuracy and may serve as a convenient and cost-effective aid for individualized risk assessment and treatment planning in clinical practice. Our findings highlight the potential of simple blood-based biomarkers to improve personalized management for patients with this rare and challenging disease.

## 1. Introduction

Small cell neuroendocrine carcinoma of the cervix (SCNEC) is a rare and highly aggressive subtype of cervical cancer, accounting for less than 2% of all cervical malignancies [1,2]. Compared to more common histologic types such as squamous cell carcinoma and adenocarcinoma, SCNEC is characterized by an increased propensity for lymphovascular invasion, lymph node metastasis, and early dissemination [3,4]. These features contribute to its dismal prognosis, with a reported 5-year overall survival (OS) rate of less than 30% [5,6,7]. Despite advancements in multimodal therapeutic approaches, including radical surgery, chemotherapy, and radiotherapy, the clinical management of SCNEC remains challenging due to diagnostic delays, limited consensus on treatment protocols, and a paucity of reliable prognostic biomarkers [8]. Common pathological markers of neuroendocrine tumors, such as chromogranin A (CgA), synaptophysin (Syn), CD56 (neural cell adhesion molecule), and neuron-specific enolase (NSE), are frequently used as ancillary tools to support pathological diagnosis [9]. Currently, prognostic factors based solely on clinicopathological characteristics are often retrospective in nature and lack real-time predictive value [10,11,12]. Therefore, there is a pressing demand for developing biomarkers and prognostic factors that can effectively assess the efficacy of treatments and enable the customization of individualized therapeutic strategies.

The systemic inflammatory response is crucial in tumor development and progression across various malignancies, with emerging evidence highlighting the prognostic value of inflammatory biomarkers in predicting anti-tumor immune responses, cancer progression, and patient survival [13,14,15]. Among inflammatory biomarkers derived from complete blood cell (CBC) count, including neutrophil-to-lymphocyte ratio (NLR), monocyte-to-lymphocyte ratio (MLR), platelet-to-lymphocyte ratio (PLR), systemic immune-inflammation index (SII = platelet count × NLR), and systemic-inflammatory-response index (SIRI = monocyte count × NLR), numerous studies have demonstrated their predictive value for patient survival, treatment response, and disease recurrence [16,17,18]. However, the prognostic utility of these systemic inflammatory biomarkers in SCNEC has not been well established. Integrating these biomarkers into prognostic and predictive models could enhance the stratification and management of patients with indeterminate risk.

This study aimed to compare the prognostic value of existing systemic inflammatory biomarkers and identify the optimal biomarker for patients with SCNEC. By investigating clinical predictors and systemic inflammatory biomarkers, it seeks to address unmet needs and develop a robust prognostic model for SCNEC.

## 2. Materials and Methods

### 2.1. Study Population

A retrospective cohort study was conducted from multiple Chinese medical centers (including Fujian Provincial Cancer Hospital, Sun Yat-Sen Memorial Hospital of Sun Yat-Sen University) that collected data on patients with SCNEC from January 2004 to June 2024. This study was approved by the medical ethical committee review board of the Fujian Cancer Hospital (K2024-206-01). The study protocol was conducted in accordance with the Declaration of Helsinki and received ethics approval with waiver of informed consent for retrospective analysis of anonymized data. Patients were enrolled based on the following inclusion criteria: age 18 years or older, had histologically confirmed SCNEC, and had complete clinical data. The exclusion criteria: (1) Hematological disorders or other systemic diseases affecting blood counts. (2) Primary tumors in other anatomical sites. (3) Clinically diagnosed infectious diseases or chronic inflammatory conditions. (4) Systemic corticosteroid or immunosuppressive therapy at the time of diagnosis. (5) Patients who could not be followed up with or had missing key clinical or laboratory data. Ultimately, 196 patients with SCNEC were included after applying the inclusion and exclusion criteria.

### 2.2. Data Collection

Various clinical variables were retrospectively collected from the medical records, including age, International Federation of Gynecology and Obstetrics (FIGO) 2018 clinical stage, neoadjuvant therapy, surgical procedure, postoperative adjuvant therapy and routine laboratory parameters. The CBCs, including leukocyte count, neutrophil count, lymphocyte count, monocyte count and platelet count, were measured within three days before the first treatment. From these measurements, several ratios were calculated, including NLR, MLR, PLR, SII, and SIRI. Progression-free survival (PFS) and overall survival (OS) were defined as the time from SCNEC diagnosis to disease progression or death, and to death from any cause, respectively, with patients without events censored at last follow-up. For patients who remained free from disease progression or had not succumbed to death by the July 2024 data cut-off, their last recorded follow-up timestamp was utilized for right-censoring in the analysis.

### 2.3. Cox Regression and Survival Analysis

Univariable and multivariable survival analyses were performed using the Cox proportional hazards model. All candidate variables—including age, tumor size, FIGO 2018 stage, neoadjuvant therapy, surgery, radiation therapy, NLR, MLR, PLR, SII, and SIRI—were first assessed in univariable Cox regression. Variables with statistically significant associations (*p* < 0.05) were subsequently included in the multivariable model. Hazard ratios (HRs) with 95% confidence intervals (CIs) were reported. The predictive performance of inflammatory biomarkers for PFS and OS was evaluated using conventional receiver operating characteristic (ROC) curve analysis, with the area under the curve (AUC) reflecting discriminatory ability. Survival outcomes were treated as binary endpoints based on status at the last follow-up. Continuous predictors were categorized after evaluation with restricted cubic splines (RCS) to relax linearity assumptions with progression risks. To explore potential nonlinear associations between SHR and patient outcomes, RCS analyses were performed using four knots placed at the 5th, 35th, 65th, and 95th percentiles. The cutoff values were determined by identifying the inflection point where the HR curve crossed 1.0. To reduce confounding due to imbalanced baseline characteristics between groups, inverse probability of treatment weighting (IPTW) based on propensity scores was applied. Propensity scores were calculated via logistic regression models, incorporating multiple covariates as independent variables and the dichotomized biomarker groups as the dependent variable. Standardized mean difference (SMD) was used to evaluate covariate balance before and after weighting, with an SMD < 0.10 indicating adequate balance. Kaplan–Meier survival curves and log-rank tests were used to compare survival distributions between groups before and after IPTW adjustment.

### 2.4. Nomogram Model Construction and Validation

The study population was randomly divided into training and test cohorts at a ratio of approximately 7:3, ensuring baseline comparability between the two groups based on predefined criteria. Prognostic factors with statistical significance (*p* < 0.05) in univariate Cox regression analysis were further entered into multivariate Cox regression to identify independent predictors, which were subsequently used to construct the nomogram. The predictive performance of the nomogram was evaluated using the concordance index (C-index). Time-dependent ROC curve analysis was conducted to determine the AUC, sensitivity, and specificity of the model in both cohorts. Calibration curves were plotted to assess the agreement between predicted and observed outcomes, while decision curve analysis (DCA) was performed to evaluate the clinical utility and net benefit of the model across a range of threshold probabilities in the training and test cohorts.

### 2.5. Statistical Analyses

All statistical analyses were performed using R software (version 4.4.1). Continuous variables were presented as mean ± standard deviation (SD), median with interquartile range (IQR), or median (range), as appropriate. Comparisons between groups for continuous variables were conducted using Student’s *t*-test or the Mann–Whitney U test, depending on the data distribution. Categorical variables were expressed as counts (percentages) and compared using the Chi-square test or Fisher’s exact test, as appropriate. A *p*-value < 0.05 was considered statistically significant.

## 3. Results

### 3.1. Patient Characteristics

Based on the inclusion and exclusion criteria, 196 patients were ultimately enrolled in this study (Figure S1). The demographic and clinical characteristics of the patients are presented in Table 1. The median age was 48 years (interquartile range [IQR]: 42.00–54.00). Tumor size was classified as ≤4 cm in 45% of patients and >4 cm in 55%. According to the 2018 FIGO staging system, patients were distributed as follows: 15% stage I, 36% stage II, 36% stage III, and 13% stage IV. Regarding treatment modalities, 26% of patients received neoadjuvant therapy, while 74% did not; 56% underwent surgical intervention, while 44% did not; and 64% received radiation therapy, while 36% did not. The median values of CBC-derived inflammatory biomarkers were as follows: NLR, 2.11 (IQR: 1.61–2.83); MLR, 0.20 (IQR: 0.15–0.28); PLR, 145.77 (IQR: 113.80–190.32); SII, 582.03 (IQR: 398.63–840.62); and SIRI, 0.76 (IQR: 0.51–1.24).

### 3.2. Survival Analysis

Patients were followed up with for a period ranging from 0 to 207 months (mean: 44.76 months, median: 22 months, IQR: 8.75–65.25). Approximately 18% of patients were followed up with for more than 100 months, a proportion unlikely to bias the survival analysis. All 196 patients were included in univariate and multivariate analyses to determine predictors of survival.

#### 3.2.1. Univariate and Multivariate Cox Regression Analysis of PFS in SCNEC Patients

Cox regression analysis was performed to identify factors influencing PFS in patients with SCNEC. Univariate analysis demonstrated that age, FIGO 2018 stage, surgery, NLR, MLR, PLR, SII, and SIRI were all significantly associated with PFS (*p* < 0.05). Variables with statistical significance (*p* < 0.05) in the univariate analysis were included in the multivariate model. The multivariate analysis identified age, FIGO 2018 stage, surgery, NLR, and MLR as independent prognostic factors for PFS in SCNEC patients (all *p* < 0.05) (Table 2).

#### 3.2.2. Univariate and Multivariate Cox Regression Analysis of OS in SCNEC Patients

Cox regression analysis was conducted to determine factors influencing OS in SCNEC patients. Univariate analysis revealed significant associations between OS and age, FIGO 2018 stage, surgery, MLR, PLR, and SIRI (all *p* < 0.05). Variables with significance (*p* < 0.05) in the univariate model were further assessed in the multivariate analysis. The multivariate analysis confirmed that age, FIGO 2018 stage, and surgery were independent prognostic factors for OS in SCNEC patients (all *p* < 0.05) (Table 2).

#### 3.2.3. Predictive Performance of Inflammatory Markers

**Figure 1:**
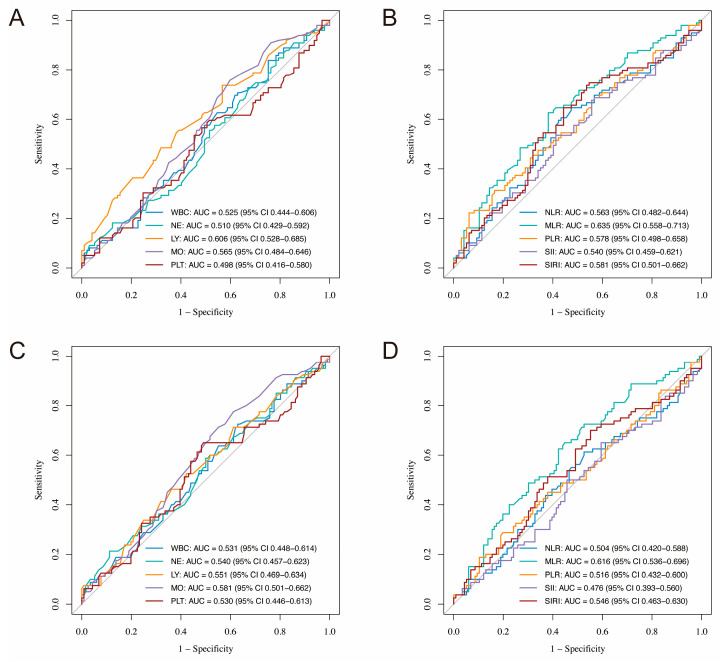
ROC curves of CBC-derived inflammatory markers for predicting prognosis in SCNEC patients. (**A**,**B**) Predictive performance for PFS; (**C**,**D**) Predictive performance for OS. ROC, receiver operating characteristic; CBC, complete blood cell; SCNEC, small cell neuroendocrine carcinoma of the cervix; PFS, progression-free survival; OS, overall survival; AUC, area under the curve; WBC, white blood cell; NE, neutrophil; LY, lymphocyte; MO, monocyte; PLT, platelet; NLR, neutrophil-to-lymphocyte ratio; MLR, monocyte-to-lymphocyte ratio; PLR, platelet-to-lymphocyte ratio; SII, systemic immune-inflammation index; SIRI, systemic-inflammatory-response index.

For PFS, the AUCs for leukocyte count, neutrophil count, lymphocyte count, monocyte count, and platelet count were 0.525, 0.510, 0.606, 0.565, and 0.498, respectively, indicating that lymphocyte and monocyte counts had relatively better predictive value compared with the other parameters (Figure 1A). Regarding OS, the corresponding AUCs were 0.531, 0.540, 0.551, 0.581, and 0.530, suggesting modestly higher predictive value of lymphocyte and monocyte counts for OS as well (Figure 1C). Among the CBC-derived inflammatory indexes, the AUCs for NLR, MLR, PLR, SII, and SIRI in predicting PFS were 0.563, 0.635, 0.578, 0.540, and 0.581, respectively. MLR showed the highest predictive performance, followed by SIRI and PLR (Figure 1B). For OS, the AUCs were 0.504, 0.616, 0.516, 0.476, and 0.546, respectively, indicating generally poor prognostic performance across all markers, though MLR remained the most predictive with the highest AUC (Figure 1D).

#### 3.2.4. Relationship Between Inflammatory Biomarker Levels and PFS

Cutoff values for NLR, MLR, PLR, SII, and SIRI, determined by RCS analysis, were 2.11, 0.19, 145.67, 587.63, and 0.75, respectively, in relation to PFS (Figure S2). Patients with elevated NLR, MLR, and PLR exhibited significantly worse PFS (*p* = 0.021, 0.0018, and 0.024, respectively; Figure S3A–C). However, SII and SIRI did not show statistically significant prognostic value for PFS (*p* = 0.066 and 0.068, respectively; Figure S3D–E).

**Figure 2:**
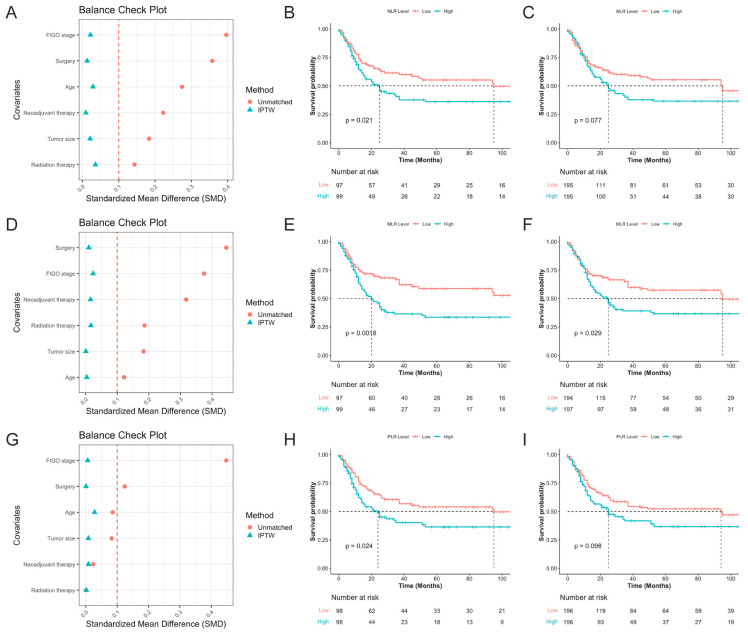
Balance check and Kaplan–Meier survival analysis for NLR, MLR, and PLR groups before and after IPTW adjustment. (**A**–**C**) NLR group—(**A**) SMD before and after IPTW; (**B**) KM survival curve before IPTW; (**C**) KM survival curve after IPTW; (**D**–**F**) MLR group—(**D**) SMD before and after IPTW; (**E**) KM survival curve before IPTW; (**F**) KM survival curve after IPTW; (**G**–**I**) PLR group—(**G**) SMD before and after IPTW; (**H**) KM survival curve before IPTW; (**I**) KM survival curve after IPTW. NLR, neutrophil-to-lymphocyte ratio; MLR, monocyte-to-lymphocyte ratio; PLR, platelet-to-lymphocyte ratio; IPTW, inverse probability of treatment weighting; SMD, standardized mean difference; KM, Kaplan–Meier.

All 196 patients were stratified into high and low groups based on NLR, MLR, and PLR levels. The corresponding baseline characteristics are presented in Table S1 (NLR), Table S2 (MLR), and Table S3 (PLR). Balance check plots for the three biomarkers before and after IPTW are shown in Figure 2A (NLR), Figure 2D (MLR), and Figure 2G (PLR), respectively. After IPTW adjustment, all baseline covariates achieved an SMD of ≤0.1, indicating successful elimination of baseline imbalances. The Kaplan–Meier survival analysis after IPTW demonstrated that patients with high MLR had significantly poorer PFS compared to those with low MLR (*p* = 0.029; Figure 2F). In contrast, post-IPTW analyses showed that neither NLR (*p* = 0.077; Figure 2C) nor PLR (*p* = 0.098; Figure 2I) retained significant prognostic value for PFS, although there were visible trends before weighting (Figure 2B,H). These findings suggest that among the three inflammatory biomarkers, MLR may be a more stable and reliable predictor of prognosis in patients with SCNEC.

As inflammatory markers were not independently associated with OS in the multivariate analysis, cutoff determination and subsequent modeling were performed only for PFS, where these indices demonstrated significant prognostic relevance.

#### 3.2.5. Subgroup Analysis

Subgroup analyses were conducted to evaluate the consistency of the association between surgical intervention and PFS across various clinically relevant strata (Figure S4). A significant interaction was observed between tumor size and surgical intervention (*p* for interaction = 0.011), indicating that tumor size modifies the effect of surgery on PFS. Among patients with tumor size ≤ 4 cm, surgical intervention was associated with a substantial reduction in the risk of progression (HR = 0.17, 95% CI: 0.09–0.32), while in those with tumors > 4 cm, the benefit was comparatively attenuated (HR = 0.56, 95% CI: 0.32–0.96). For the remaining subgroups—including age, FIGO stage, neoadjuvant therapy, radiation therapy, and inflammatory marker levels—no significant interactions were observed (all *p* for interaction > 0.1), suggesting that the association between surgical intervention and PFS was generally consistent across these subpopulations.

### 3.3. Construction and Validation of Nomogram

#### 3.3.1. Patient Cohort Division

All patients were randomly divided into training cohort (137 cases) and test cohort (59 cases). There were no statistically significant differences in baseline characteristics between the two cohorts (*p* > 0.05) (Table S4).

#### 3.3.2. Feature Selection

In the univariate Cox analysis, eight variables were found to be associated with PFS of patients with SCNEC, including age, FIGO 2018 stage, surgery, NLR, MLR, PLR, SII, and SIRI (all *p* < 0.05). Then, multivariate Cox analysis was performed and four variables were finally determined as independent prognostic factors, including age, FIGO 2018 stage, surgery, NLR, and MLR (Table 2). RCS analysis identified optimal prognostic cutoffs for NLR (2.11) and MLR (0.19), with both parameters showing significant survival stratification in Kaplan–Meier analysis (*p* < 0.05). However, IPTW revealed differential stability: MLR retained significant prognostic discrimination post-adjustment (*p* = 0.029), whereas NLR lost statistical significance (*p* = 0.077). Consequently, the definitive model incorporated age, FIGO 2018 stage, surgery, and MLR-based stratification.

#### 3.3.3. Nomogram Construction

**Figure 3:**
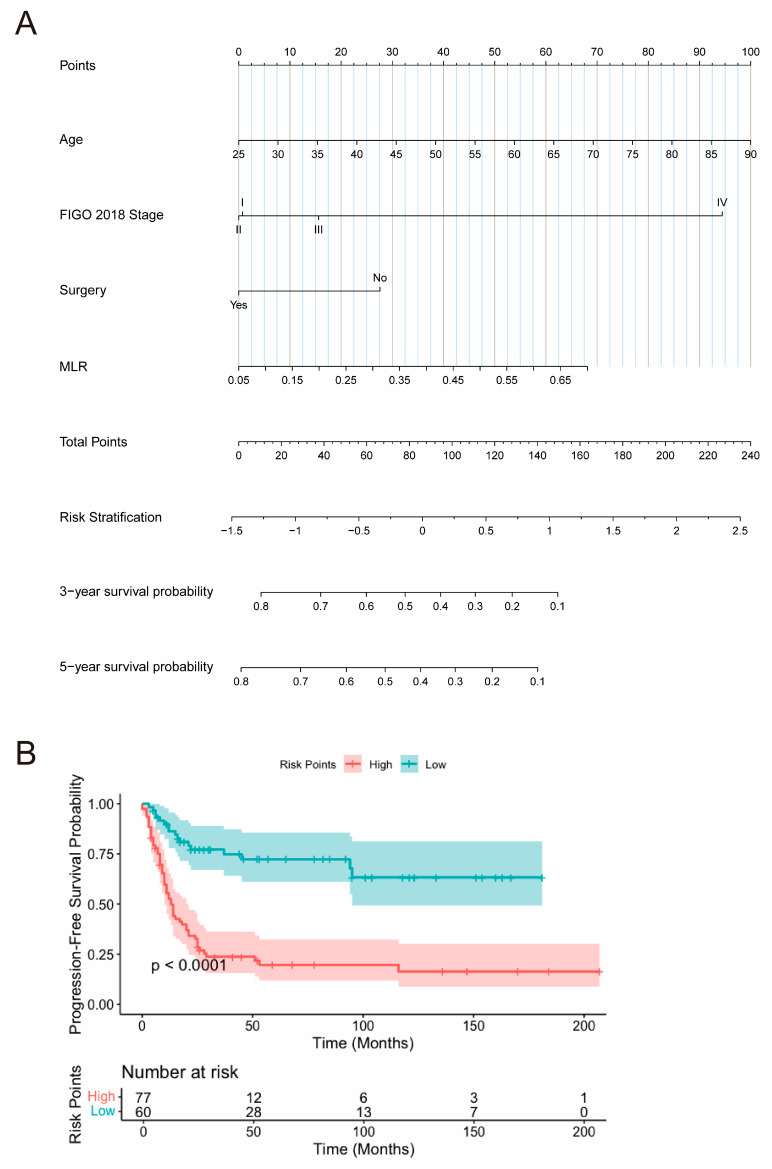
(**A**) Nomogram to predict the probability of PFS in SCNEC patients; (**B**) Kaplan–Meier survival curves for risk stratification based on total points. PFS, progression-free survival; SCNEC, small cell neuroendocrine carcinoma of the cervix; FIGO, International Federation of Gynecology and Obstetrics; MLR, monocyte-to-lymphocyte ratio.

A nomogram was developed based on these independent prognostic factors to predict 3-year and 5-year PFS in SCNEC patients (Figure 3A). Patients with younger age, early-stage FIGO 2018 classification, a history of surgical treatment, and lower MLR levels demonstrated better PFS outcomes. Furthermore, a total risk score for each patient was calculated using the nomogram, allowing stratification into low-risk (total score ≤ 67.03) and high-risk (total score > 67.03) subgroups. Kaplan–Meier survival analysis, along with the log-rank test, revealed a statistically significant difference between the survival curves of these two subgroups in both the training and test cohorts (*p* < 0.0001) (Figure 3B). Patients in the high-risk group exhibited significantly worse prognoses compared to those in the low-risk group.

#### 3.3.4. Nomogram Validation

**Figure 4:**
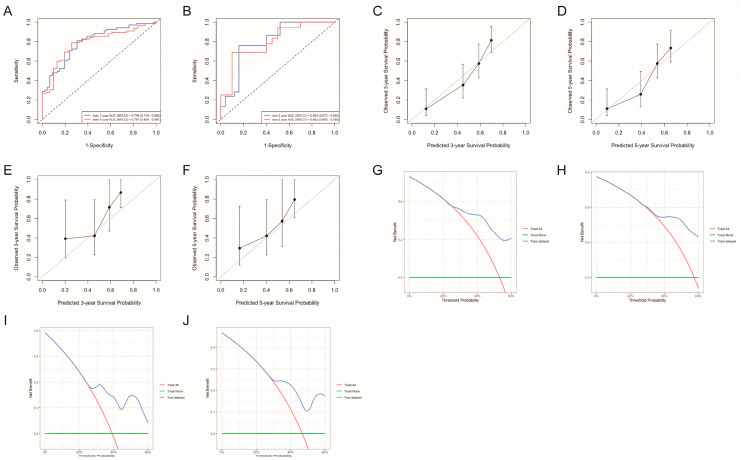
The predictive value of the nomogram in SCNEC patients. (**A**,**B**) ROC curves for the training and test cohorts, respectively; (**C**,**D**) Calibration curves for 3-year and 5-year PFS in the training cohort; (**E**,**F**) Calibration curves for 3-year and 5-year PFS in the test cohort; (**G**,**H**) DCA for 3-year and 5-year PFS in the train cohort; (**I**,**J**) DCA for 3-year and 5-year PFS in the test cohort, demonstrating the clinical utility of the nomogram. SCNEC, small cell neuroendocrine carcinoma of the cervix; ROC, receiver operating characteristic; AUC, area under the curve; PFS, progression-free survival.

The predictive performance of the nomogram was evaluated, yielding a C-index of 0.725 in the training cohort and 0.705 in the test cohort, indicating robust discriminative ability. Additionally, ROC curve analysis demonstrated strong predictive accuracy, with AUC values of 0.799 (95% CI: 0.718–0.880) and 0.787 (95% CI: 0.694–0.881) for 3-year and 5-year PFS in the training cohort (Figure 4A), and 0.802 (95% CI: 0.673–0.900) and 0.802 (95% CI: 0.669–0.905) in the test cohort (Figure 4B), respectively. Calibration curves for 3-year and 5-year PFS exhibited excellent agreement between predicted and observed survival rates (Figure 4C–F). Furthermore, DCA confirmed the clinical utility of the PFS nomogram (Figure 4G–J). These findings collectively indicate that the nomogram is a reliable and accurate tool for predicting the prognosis of SCNEC patients.

## 4. Discussion

This retrospective study comprehensively evaluated the prognostic value of CBC-derived inflammatory biomarkers in patients with SCNEC. Elevated NLR, MLR, and PLR were associated with worse PFS, among which MLR showed the highest prognostic performance and retained significance after IPTW, highlighting its robustness. In addition, age, FIGO 2018 stage, and surgical intervention emerged as independent predictors for both PFS and OS. A nomogram incorporating age, stage, surgery, and MLR demonstrated good discrimination (C-index: 0.725 in training and 0.705 in test cohorts) and calibration, supporting its potential utility in risk-adapted management.

Uncontrolled inflammation is widely believed to be closely associated with tumor initiation, progression, invasion, and metastasis [14,19,20]. Inflammatory cells are key components of the tumor microenvironment [21], and their counts can be assessed through routine laboratory tests. Lymphocytes play a crucial role in the immune response by contributing to tumor surveillance and defense [22]. Neutrophils, as first responders to inflammation, have been increasingly recognized for their involvement in tumor progression and cancer development [23]. Furthermore, platelets have been shown to promote tumor growth and metastasis [24]. The prognostic role of systemic inflammation in malignancies has been increasingly recognized, and our results align with this growing body of evidence [18,25,26,27,28,29]. Inflammation-related indices such as NLR, MLR, PLR, SII, and SIRI reflect the balance between pro-tumor immune suppression and anti-tumor immune responses. Among them, MLR demonstrated the most stable prognostic value in our SCNEC cohort. This may be due to the dual role of monocytes in promoting tumor progression through angiogenesis and immunosuppression [30], while lymphocytes are essential for anti-tumor immunity [22]. Although NLR and PLR were also associated with PFS in unadjusted analysis, they lost significance after IPTW, suggesting their prognostic value may be confounded by baseline covariates. The superior performance of MLR in both multivariate Cox and IPTW-adjusted Kaplan–Meier analyses underscores its potential as a more reliable biomarker in this rare and aggressive disease. Therefore, a high MLR could reflect an imbalance favoring tumor-promoting inflammation over anti-tumor immune responses.

While FIGO stage and age were confirmed as prognostic factors consistent with previous studies [12], benefit of surgical intervention demonstrated significant dependency on tumor size. Subgroup analysis revealed that tumor size significantly modified the impact of surgery on PFS, with patients harboring tumors ≤4 cm deriving notably greater benefit compared to those with larger tumors. This finding highlights the critical role of tumor burden in determining the therapeutic value of surgical resection and supports the use of tumor size as a stratification factor in treatment decision-making for SCNEC. It also aligns with earlier reports identifying tumor size as a key prognostic indicator in cervical cancer [31]. In contrast, other subgroup factors such as age, stage, and inflammatory biomarker levels did not exhibit significant interaction with surgical benefit, indicating the broadly consistent benefit of surgery across most patient subgroups.

The construction of a nomogram incorporating age, FIGO 2018 stage, surgical intervention, and MLR represents a major outcome of this study. This tool enables individualized prediction of 3- and 5-year PFS, demonstrating good discrimination and calibration. Both training and test cohorts achieved C-indices exceeding 0.70, indicating robust internal validity. DCA further confirmed the model’s net clinical benefit across a range of threshold probabilities. Compared with previous models relying solely on clinical parameters [32], our integrated approach—combining clinical and inflammatory markers—yielded superior predictive accuracy. Notably, the AUCs for predicting 3- and 5-year PFS in the test cohort were both 0.802, indicating superior performance compared to individual inflammatory markers. Clinically, the MLR-enhanced nomogram may provide clinically useful risk stratification and can facilitate real-time prognostication without incurring additional costs, which is particularly advantageous in resource-limited settings. Given the aggressive nature of SCNEC and the constraints on access to molecular testing, this nomogram may serve as a practical, bedside-ready tool for guiding individualized management. While OS is generally regarded as the most definitive endpoint, PFS was selected for nomogram construction because inflammatory biomarkers demonstrated independent prognostic value for PFS but not for OS in the multivariate analysis. Furthermore, given the aggressive course of SCNEC and the relatively short follow-up period for many patients, PFS provided a more sensitive and timely reflection of disease progression and treatment efficacy in this cohort.

Although our study adopted a multicenter design and included a relatively large cohort for a rare malignancy such as SCNEC, several limitations should be acknowledged. First, the training and test cohorts originated from the same source population via stratified random sampling. While this approach ensured internal consistency, future validation in external cohorts is warranted to further reinforce the model’s generalizability. Second, the assessment of MLR at a single time point may fail to capture dynamic fluctuations over the disease course, underscoring the need for future studies incorporating serial monitoring. Third, the absence of paired tissue-based immune profiling limits our ability to establish mechanistic links between peripheral MLR and tumor-infiltrating immune cells, particularly tumor-associated macrophages. This limitation could be addressed in future investigations utilizing transcriptomic or spatial profiling approaches.

In addition, while the retrospective nature of the study inherently restricts causal inferences, the use of IPTW helps to mitigate immortal time bias more effectively than traditional multivariable adjustments. In future studies, prospective validation using independent cohorts will be essential to confirm our findings. Moreover, the integration of multi-omics data—including proteomics and transcriptomics—may facilitate the discovery of novel biomarkers and therapeutic targets. Finally, deeper exploration of the interplay between systemic inflammatory responses and the tumor microenvironment may enhance our understanding of SCNEC pathobiology and support the development of individualized therapeutic strategies.

## 5. Conclusions

This study enhances the understanding of SCNEC prognosis by integrating clinical characteristics and inflammatory biomarkers, providing a novel approach to risk stratification. Our findings have the potential to contribute to more precise clinical decision-making and may serve as a foundation for further research aimed at optimizing individualized patient management in this domain.

## Figures and Tables

**Table 1:** Baseline variables of adults with CBC-derived inflammatory biomarkers.

Characteristic	N = 196 ^1^
Age	48.00 (42.00, 54.00)
Tumor size	
≤4 cm	88 (45%)
>4 cm	108 (55%)
FIGO 2018 stage	
I	30 (15%)
II	71 (36%)
III	70 (36%)
IV	25 (13%)
Neoadjuvant therapy	
No	145 (74%)
Yes	51 (26%)
Surgery	
No	86 (44%)
Yes	110 (56%)
Radiation therapy	
No	70 (36%)
Yes	126 (64%)
CBC count, 10^3^/uL	
White blood cell	6.34 (5.39, 8.00)
Neutrophils	3.91 (3.10, 5.20)
Lymphocyte	1.90 (1.50, 2.25)
Monocyte	0.39 (0.29, 0.50)
Platelet	276.50 (227.00, 334.00)
CBC-derived indicators	
NLR	2.11 (1.61, 2.83)
MLR	0.20 (0.15, 0.28)
PLR	145.77 (113.80, 190.32)
SII	582.03 (398.63, 840.62)
SIRI	0.76 (0.51, 1.24)

^1^ Median (Q1, Q3); n (%); FIGO, International Federation of Gynecology and Obstetrics; CBC, complete blood cell; NLR, neutrophil-to-lymphocyte ratio; MLR, monocyte-to-lymphocyte ratio; PLR, platelet-to-lymphocyte ratio; SII, systemic immune-inflammation index; SIRI, systemic-inflammatory-response index.

**Table 2:** Univariate and multivariate Cox regression analysis of PFS and OS in SCNEC patients.

	PFS (Univariate Analysis)			PFS (Multivariate Analysis)			OS (Univariate Analysis)			OS (Multivariate Analysis)		
Characteristic	HR	95% CI	*p*-Value	HR	95% CI	*p*-Value	HR	95% CI	*p*-Value	HR	95% CI	*p*-Value
Age	1.03	1.01, 1.05	0.003	1.03	1.01, 1.05	0.004	1.04	1.02, 1.06	<0.001	1.04	1.01, 1.06	0.001
Tumor size			0.081						0.16			
≤4 cm	—	—					—	—				
>4 cm	1.43	0.95, 2.14					1.37	0.88, 2.15				
FIGO 2018 stage			<0.001			<0.001			<0.001			0.004
I	—	—		—	—		—	—		—	—	
II	1.94	0.86, 4.37		1.47	0.63, 3.45		2.93	1.03, 8.31		2.07	0.70, 6.13	
III	2.68	1.19, 6.03		1.87	0.80, 4.39		3.31	1.16, 9.47		2.16	0.72, 6.47	
IV	11.6	4.88, 27.5		5.98	2.22, 16.1		16.2	5.45, 48.0		6.12	1.84, 20.3	
Neoadjuvant therapy			0.084						0.13			
No	—	—					—	—		—	—	
Yes	0.67	0.41, 1.07					0.68	0.41, 1.14				
Surgery			<0.001			0.020			<0.001			0.014
No	—	—		—	—		—	—		—	—	
Yes	0.36	0.24, 0.53		0.56	0.35, 0.91		0.30	0.19, 0.47		0.51	0.30, 0.87	
Radiation therapy			0.47						0.59			
No	—	—					—	—				
Yes	0.86	0.57, 1.29					0.88	0.56, 1.39				
NLR	1.19	1.05, 1.34	0.016	0.68	0.45, 1.01	0.050	1.15	0.98, 1.36	0.11			
MLR	19.6	3.95, 97.2	<0.001	1.01	1.00, 1.01	0.005	12.5	2.08, 75.3	0.009	1.68	0.07, 42.0	0.75
PLR	1.00	1.00, 1.01	<0.001	3.04	0.12, 79.1	0.51	1.00	1.00, 1.01	0.050	1.00	1.00, 1.01	0.13
SII	1.00	1.00, 1.00	0.016	1.00	1.00, 1.00	0.89	1.00	1.00, 1.00	0.071			
SIRI	1.27	1.09, 1.49	0.008	1.47	0.94, 2.30	0.12	1.30	1.08, 1.57	0.014	1.06	0.75, 1.52	0.74

PFS, progression-free survival; OS, overall survival; SCNEC, small cell neuroendocrine carcinoma of the cervix; HR, Hazard Ratio; CI, Confidence Interval; FIGO, International Federation of Gynecology and Obstetrics; NLR, neutrophil-to-lymphocyte ratio; MLR, monocyte-to-lymphocyte ratio; PLR, platelet-to-lymphocyte ratio; SII, systemic immune-inflammation index; SIRI, systemic-inflammatory-response index.

## Data Availability

The data supporting the results of this study are available from the corresponding authors upon reasonable request.

## Supplementary Materials

The following supporting information can be downloaded at: https://www.mdpi.com/article/10.3390/curroncol32120654/s1, Table S1: Baseline characteristics of patients grouped by NLR before and after adjustment using IPTW; Table S2: Baseline characteristics of patients grouped by MLR before and after adjustment using IPTW; Table S3: Baseline characteristics of patients grouped by PLR before and after adjustment using IPTW; Table S4: Comparison of factor characteristics between training cohort and test cohort. Figure S1: Study profile; Figure S2: RCS curves for determining the optimal cutoff values of continuous variables in SCNEC patients, including age (A), NLR (B), MLR (C), PLR (D), SII (E), and SIRI (F); Figure S3: Kaplan–Meier survival curves for PFS in SCNEC patients, showing the prognostic impact of NLR (A), MLR (B), PLR (C), SII (D), and SIRI (E); Figure S4: Subgroup analysis of the association between surgical intervention and PFS in SCNEC patients.

## References

[1] Crowder S., Tuller E. (2007). Small Cell Carcinoma of the Female Genital Tract. Semin. Oncol..

[2] Chao A., Wu R.-C., Lin C.-Y., Chang T.-C., Lai C.-H. (2023). Small Cell Neuroendocrine Carcinoma of the Cervix: From Molecular Basis to Therapeutic Advances. Biomed. J..

[3] McCusker M.E., Coté T.R., Clegg L.X., Tavassoli F.J. (2003). Endocrine Tumors of the Uterine Cervix: Incidence, Demographics, and Survival with Comparison to Squamous Cell Carcinoma. Gynecol. Oncol..

[4] Park K.J., Soslow R.A., Nucci M.R., Parra-Herran C. (2020). Neoplastic Lesions of the Cervix. Gynecologic Pathology.

[5] Cohen J.G., Kapp D.S., Shin J.Y., Urban R., Sherman A.E., Chen L., Osann K., Chan J.K. (2010). Small Cell Carcinoma of the Cervix: Treatment and Survival Outcomes of 188 Patients. Am. J. Obstet. Gynecol..

[6] Intaraphet S., Kasatpibal N., Siriaunkgul S., Chandacham A., Sukpan K., Patumanond J. (2014). Prognostic Factors for Small Cell Neuroendocrine Carcinoma of the Uterine Cervix: An Institutional Experience. Int. J. Gynecol. Cancer.

[7] Hou W.-H., Schultheiss T.E., Wong J.Y., Wakabayashi M.T., Chen Y.-J. (2018). Surgery versus Radiation Treatment for High-Grade Neuroendocrine Cancer of Uterine Cervix: A Surveillance Epidemiology and End Results Database Analysis. Int. J. Gynecol. Cancer.

[8] Winer I., Kim C., Gehrig P. (2021). Neuroendocrine Tumors of the Gynecologic Tract Update. Gynecol. Oncol..

[9] Tempfer C.B., Tischoff I., Dogan A., Hilal Z., Schultheis B., Kern P., Rezniczek G.A. (2018). Neuroendocrine Carcinoma of the Cervix: A Systematic Review of the Literature. BMC Cancer.

[10] Zhang Q., Xiong Y., Ye J., Zhang L., Li L. (2018). Influence of Clinicopathological Characteristics and Comprehensive Treatment Models on the Prognosis of Small Cell Carcinoma of the Cervix: A Systematic Review and Meta-Analysis. PLoS ONE.

[11] Huang R., Gan Q., Cheng J. (2020). Prognostic Factors and Local Treatment Modalities of Small-Cell Carcinoma of the Cervix: An Analysis According to the International Federation of Gynecology and Obstetrics Stage. Cancer Manag. Res..

[12] Chu T., Meng Y., Wu P., Li Z., Wen H., Ren F., Zou D., Lu H., Wu L., Zhou S. (2023). The Prognosis of Patients with Small Cell Carcinoma of the Cervix: A Retrospective Study of the SEER Database and a Chinese Multicentre Registry. Lancet Oncol..

[13] Leon-Cabrera S., Schwertfeger K.L., Terrazas L.I. (2019). Inflammation as a Target in Cancer Therapy. Mediat. Inflamm..

[14] Greten F.R., Grivennikov S.I. (2019). Inflammation and Cancer: Triggers, Mechanisms, and Consequences. Immunity.

[15] Wu D.-Z., Zhong J.-M., Jiang W.-P., Liao Z.-S., Huang S.-H., Sun Y.-W., Lin Y., Ye D.-X., Pan C., Jiang W.-Z. (2022). Preoperative Combination Score of Neutrophils, Monocytes, and Lymphocytes as a Predictor for Locally Advanced Rectal Cancer. Int. J. Color. Dis..

[16] Xie H., Ruan G., Wei L., Deng L., Zhang Q., Ge Y., Song M., Zhang X., Lin S., Liu X. (2023). The Inflammatory Burden Index Is a Superior Systemic Inflammation Biomarker for the Prognosis of Non-Small Cell Lung Cancer. J. Cachexia Sarcopenia Muscle.

[17] Xu N., Zhang J.-X., Zhang J.-J., Huang Z., Mao L.-C., Zhang Z.-Y., Jin W.-D. (2025). The Prognostic Value of the Neutrophil-to-Lymphocyte Ratio (NLR) and Platelet-to-Lymphocyte Ratio (PLR) in Colorectal Cancer and Colorectal Anastomotic Leakage Patients: A Retrospective Study. BMC Surg..

[18] Sakurai A., Yamaguchi K., Ishida K., Horikawa N., Kawai E., Kotani Y., Yoshida T., Kishimoto N., Tatsumi K., Okudate M. (2025). Prognostic Significance of Neutrophil-to-Lymphocyte Ratio, Platelet-to-Lymphocyte Ratio, and Monocyte-to-Lymphocyte Ratio in Uterine Carcinosarcoma. Int. J. Clin. Oncol..

[19] Mantovani A., Allavena P., Sica A., Balkwill F. (2008). Cancer-Related Inflammation. Nature.

[20] Coffelt S.B., de Visser K.E. (2014). Cancer: Inflammation Lights the Way to Metastasis. Nature.

[21] Yamashita H., Katai H. (2010). Systemic Inflammatory Response in Gastric Cancer. World J. Surg..

[22] Criscitiello C., Esposito A., Trapani D., Curigliano G. (2016). Prognostic and Predictive Value of Tumor Infiltrating Lymphocytes in Early Breast Cancer. Cancer Treat. Rev..

[23] Mollinedo F. (2019). Neutrophil Degranulation, Plasticity, and Cancer Metastasis. Trends Immunol..

[24] Yan M., Jurasz P. (2016). The Role of Platelets in the Tumor Microenvironment: From Solid Tumors to Leukemia. Biochim. Biophys. Acta.

[25] Li Y.-X., Chang J.-Y., He M.-Y., Wang H.-R., Luo D.-Q., Li F.-H., Li J.-H., Ran L. (2021). Neutrophil-to-Lymphocyte Ratio (NLR) and Monocyte-to-Lymphocyte Ratio (MLR) Predict Clinical Outcome in Patients with Stage IIB Cervical Cancer. J. Oncol..

[26] Domenici L., Tonacci A., Aretini P., Garibaldi S., Perutelli A., Bottone P., Muzii L., Benedetti Panici P. (2021). Inflammatory Biomarkers as Promising Predictors of Prognosis in Cervical Cancer Patients. Oncology.

[27] Li J., Cao D., Huang Y., Xiong Q., Tan D., Liu L., Lin T., Wei Q. (2022). The Prognostic and Clinicopathological Significance of Systemic Immune-Inflammation Index in Bladder Cancer. Front. Immunol..

[28] Xie Y., Yu Q., Zhu Y., Wu W., Xiao R., Wang N., Zhu L., Li P., Chen T. (2025). The Value of Peripheral Blood Inflammation Markers in Risk Assessment and Prediction of Lung Cancer. Future Sci. OA.

[29] Ma W., Liu R., Li X., Yu J., Wang W. (2025). Significant Association between Systemic Inflammation Response Index and Prognosis in Patients with Urological Malignancies. Front. Immunol..

[30] Tan L.Y., Cockshell M.P., Moore E., Myo Min K.K., Ortiz M., Johan M.Z., Ebert B., Ruszkiewicz A., Brown M.P., Ebert L.M. (2022). Vasculogenic Mimicry Structures in Melanoma Support the Recruitment of Monocytes. Oncoimmunology.

[31] Horn L.-C., Fischer U., Raptis G., Bilek K., Hentschel B. (2007). Tumor Size Is of Prognostic Value in Surgically Treated FIGO Stage II Cervical Cancer. Gynecol. Oncol..

[32] Xie N., Yu H., Lin J., Deng S., Liu L., Sun Y. (2025). A Nomogram for Predicting Prognosis for Young Cervical Neuroendocrine Carcinoma: A SEER-Based Study and External Validation. Front. Oncol..

