# Supplementary material for: Prognostic Significance of Complete Blood Count-Derived Inflammatory Biomarkers in Patients with Small Cell Neuroendocrine Carcinoma of the Cervix
Source: Curr Oncol. 2025 Nov 21;32(12):654. doi: 10.3390/curroncol32120654 (PMC12731553; doi:10.3390/curroncol32120654)
Supplement: Supplementary file 1 [file curroncol-32-00654-s001.zip › curroncol-3858498-supplementary.pdf]

Table S1 Baseline characteristics of patients grouped by NLR before and after adjustment using IPTW

	**Level**	**Unmatched**				**IPTW**			
**Group**	**Characteristics**	**Low NLR**	**High NLR**	***p***	**SMD**	**Low NLR**	**High NLR**	***p***	**SMD**
N		97	99			195.49	194.7		
Age (mean (SD))		50.02 (11.80)	47.03 (9.93)	0.06	0.27	48.51 (11.65)	48.20 (9.82)	0.85	0.03
Tumor size (%)	≤4 cm	48 (49.5)	40 (40.4)	0.2	0.18	88.6 (45.3)	86.2 (44.3)	0.89	0.02
	>4 cm	49 (50.5)	59 (59.6)			106.9 (54.7)	108.5 (55.7)		
FIGO stage (%)	I	14 (14.4)	16 (16.2)	0.06	0.4	32.2 (16.5)	31.2 (16.0)	1	0.02
	II	42 (43.3)	29 (29.3)			72.5 (37.1)	71.5 (36.7)		
	III	34 (35.1)	36 (36.4)			67.0 (34.3)	67.2 (34.5)		
	IV	7 (7.2)	18 (18.2)			23.8 (12.2)	24.8 (12.8)		
Neoadjuvant therapy (%)	No	67 (69.1)	78 (78.8)	0.12	0.22	144.5 (73.9)	143.1 (73.5)	0.95	0.01
	Yes	30 (30.9)	21 (21.2)			51.0 (26.1)	51.6 (26.5)		
Surgery (%)	No	34 (35.1)	52 (52.5)	0.01	0.36	85.5 (43.7)	86.4 (44.4)	0.93	0.01
	Yes	63 (64.9)	47 (47.5)			110.0 (56.3)	108.3 (55.6)		
Radiation therapy (%)	No	38 (39.2)	32 (32.3)	0.32	0.14	72.2 (37.0)	68.6 (35.2)	0.82	0.04
	Yes	59 (60.8)	67 (67.7)			123.3 (63.0)	126.1 (64.8)		

NLR, neutrophil-to-lymphocyte ratio; IPTW, inverse probability of treatment weighting; SMD, standardized mean difference; FIGO, International Federation of Gynecology and Obstetrics.

Table S2 Baseline characteristics of patients grouped by MLR before and after adjustment using IPTW

	Level	Unmatched				IPTW			
**Group**	**Characteristics**	**Low MLR**	**High MLR**	***p***	**SMD**	**Low MLR**	**High MLR**	***p***	**SMD**
N		97	99			194.36	196.97		
Age (mean (SD))		49.19 (11.08)	47.85 (10.87)	0.395	0.122	48.97 (11.10)	49.01 (11.42)	0.981	0.004
Tumor size (%)	≤4 cm	48 (49.5)	40 (40.4)	0.201	0.183	86.7 (44.6)	87.7 (44.5)	0.995	0.001
	>4 cm	49 (50.5)	59 (59.6)			107.7 (55.4)	109.2 (55.5)		
FIGO stage (%)	I	16 (16.5)	14 (14.1)	0.084	0.375	31.2 (16.1)	30.9 (15.7)	0.999	0.024
	II	42 (43.3)	29 (29.3)			70.0 (36.0)	71.0 (36.0)		
	III	31 (32.0)	39 (39.4)			70.4 (36.2)	70.6 (35.8)		
	IV	8 (8.2)	17 (17.2)			22.7 (11.7)	24.5 (12.4)		
Neoadjuvant therapy (%)	No	65 (67.0)	80 (80.8)	0.028	0.318	143.2 (73.7)	146.5 (74.4)	0.918	0.016
	Yes	32 (33.0)	19 (19.2)			51.2 (26.3)	50.5 (25.6)		
Surgery (%)	No	32 (33.0)	54 (54.5)	0.002	0.455	83.6 (43.0)	85.7 (43.5)	0.946	0.01
	Yes	65 (67.0)	45 (45.5)			110.8 (57.0)	111.3 (56.5)		
Radiation therapy (%)	No	39 (40.2)	31 (31.3)	0.194	0.186	70.0 (36.0)	69.4 (35.2)	0.912	0.017
	Yes	58 (59.8)	68 (68.7)			124.3 (64.0)	127.6 (64.8)		

MLR, monocyte-to-lymphocyte ratio; IPTW, inverse probability of treatment weighting; SMD, standardized mean difference; FIGO, International Federation of Gynecology and Obstetrics.

Table S3 Baseline characteristics of patients grouped by PLR before and after adjustment using IPTW

Group	Level Characteristics	Unmatched Low PLR	High PLR	*p*	SMD	IPTW Low PLR	High PLR	*p*	SMD
N		98	98			195.93	195.64		
Age (mean (SD))		48.98 (11.72)	48.04 (10.20)	0.55	0.085	48.85 (11.63)	48.55 (9.77)	0.848	0.028
Tumor size (%)	≤4 cm	46 (46.9)	42 (42.9)	0.566	0.082	89.1 (45.5)	88.2 (45.1)	0.957	0.008
	>4 cm	52 (53.1)	56 (57.1)			106.8 (54.5)	107.4 (54.9)		
FIGO stage (%)	I	14 (14.3)	16 (16.3)	0.025	0.448	30.6 (15.6)	30.4 (15.5)	1	0.006
	II	45 (45.9)	26 (26.5)			71.1 (36.3)	70.6 (36.1)		
	III	31 (31.6)	39 (39.8)			69.3 (35.4)	69.6 (35.6)		
	IV	8 (8.2)	17 (17.3)			24.9 (12.7)	25.1 (12.8)		
Neoadjuvant therapy (%)	No	72 (73.5)	73 (74.5)	0.871	0.023	143.2 (73.1)	142.3 (72.7)	0.956	0.008
	Yes	26 (26.5)	25 (25.5)			52.7 (26.9)	53.4 (27.3)		
Surgery (%)	No	40 (40.8)	46 (46.9)	0.388	0.124	84.8 (43.3)	84.7 (43.3)	0.997	0.001
	Yes	58 (59.2)	52 (53.1)			111.2 (56.7)	110.9 (56.7)		
Radiation therapy (%)	No	35 (35.7)	35 (35.7)	1	<0.001	68.2 (34.8)	68.3 (34.9)	0.991	0.002
	Yes	63 (64.3)	63 (64.3)			127.7 (65.2)	127.4 (65.1)		

PLR, platelet-to-lymphocyte ratio; IPTW, inverse probability of treatment weighting; SMD, standardized mean difference; FIGO, International Federation of Gynecology and Obstetrics.

Table S4 Comparison of factor characteristics between training cohort and test cohort

**Characteristic**	**Overall**<br>N = 196[1]	**Train**<br>N = 137[1]	**Test**<br>N = 59[1]	***p*-value**[2]
Age	48.00 (42.00, 54.00)	49.00 (41.00, 54.00)	47.00 (43.00, 53.00)	>0.9
Tumor size				0.4
≤4 cm	88 (45%)	59 (43%)	29 (49%)	
>4 cm	108 (55%)	78 (57%)	30 (51%)	
FIGO 2018 stage				0.6
I	30 (15%)	18 (13%)	12 (20%)	
II	71 (36%)	51 (37%)	20 (34%)	
III	70 (36%)	49 (36%)	21 (36%)	
IV	25 (13%)	19 (14%)	6 (10%)	
Neoadjuvant therapy				0.4
No	145 (74%)	99 (72%)	46 (78%)	
Yes	51 (26%)	38 (28%)	13 (22%)	
Surgery				0.8
No	86 (44%)	61 (45%)	25 (42%)	
Yes	110 (56%)	76 (55%)	34 (58%)	
Radiation therapy				0.2
No	70 (36%)	53 (39%)	17 (29%)	
Yes	126 (64%)	84 (61%)	42 (71%)	
NLR	2.11 (1.61, 2.83)	2.11 (1.67, 2.89)	2.10 (1.46, 2.78)	0.4
MLR	0.20 (0.15, 0.28)	0.20 (0.15, 0.28)	0.19 (0.15, 0.27)	0.7
PLR	145.77 (113.80, 190.32)	144.81 (113.47, 182.80)	147.69 (114.78, 211.30)	0.4
SII, 10E3/uL	582.03 (398.63, 840.62)	590.53 (401.39, 850.69)	537.50 (375.72, 832.70)	0.7
SIRI, 10E3/uL	0.76 (0.51, 1.24)	0.78 (0.54, 1.22)	0.71 (0.44, 1.25)	0.3

[1] Median (Q1, Q3); n (%)

[2] Wilcoxon rank sum test; Pearson's Chi-squared test

FIGO, International Federation of Gynecology and Obstetrics; NLR, neutrophil-to-lymphocyte ratio; MLR, monocyte-to-lymphocyte ratio; PLR, platelet-to-lymphocyte ratio; SII, systemic immune-inflammation index; SIRI, systemic-inflammatory-response index.

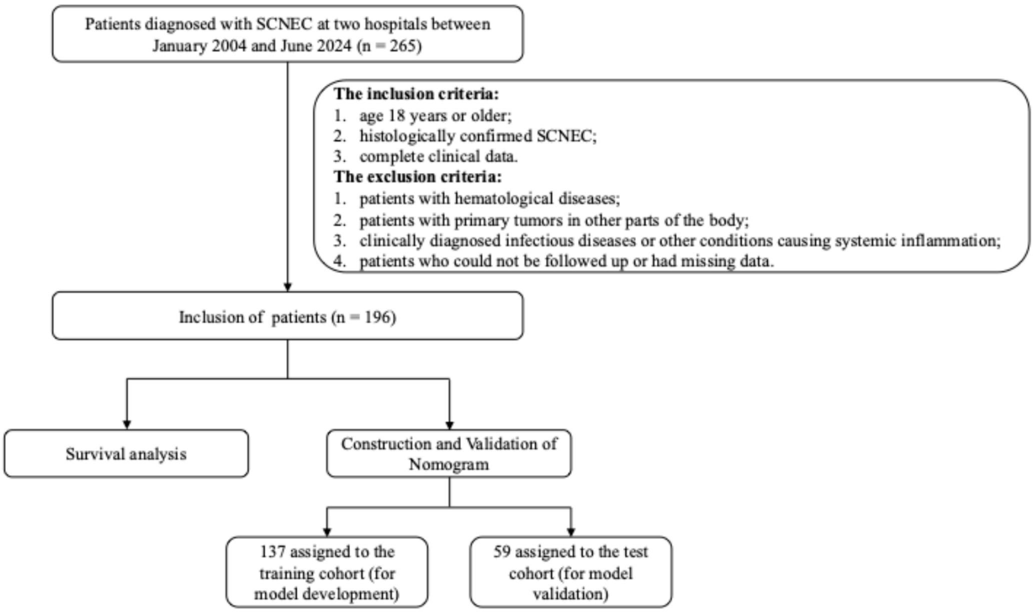

Figure S1 Study profile

SCNEC, small cell neuroendocrine carcinoma of the cervix.

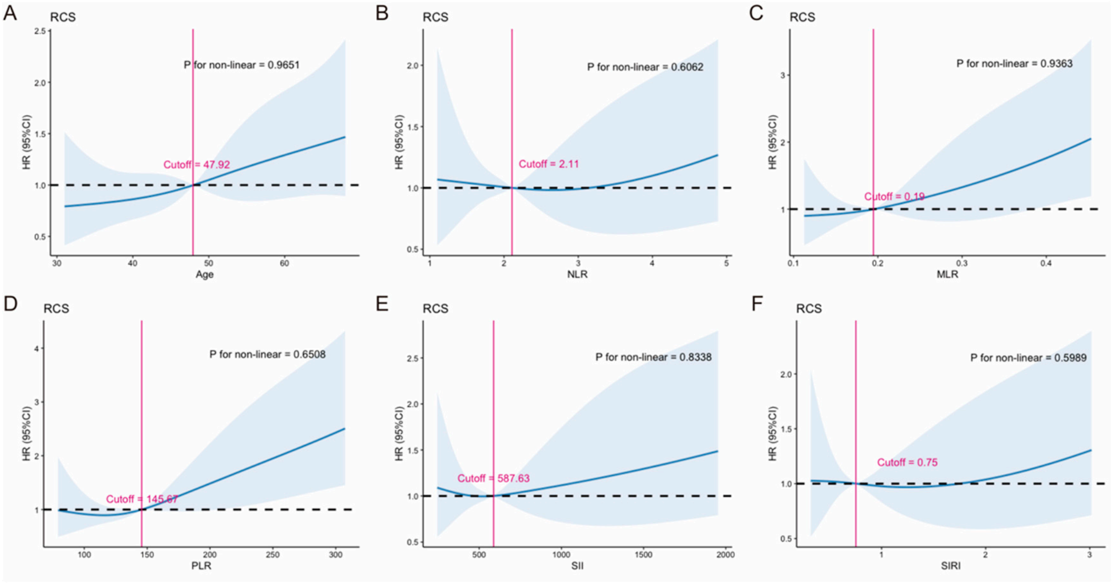

Figure S2 RCS curves for determining the optimal cutoff values of continuous variables in SCNEC patients, including age (A), NLR (B), MLR (C), PLR (D), SII (E), and SIRI (F).

RCS, restricted cubic splines; SCNEC, small cell neuroendocrine carcinoma of the cervix; NLR, neutrophil-to-lymphocyte ratio; MLR, monocyte-to-lymphocyte ratio; PLR, platelet-to-lymphocyte ratio; SII, systemic immune-inflammation index; SIRI, systemic-inflammatory-response index; HR, Hazard Ratio; CI, Confidence Interval.

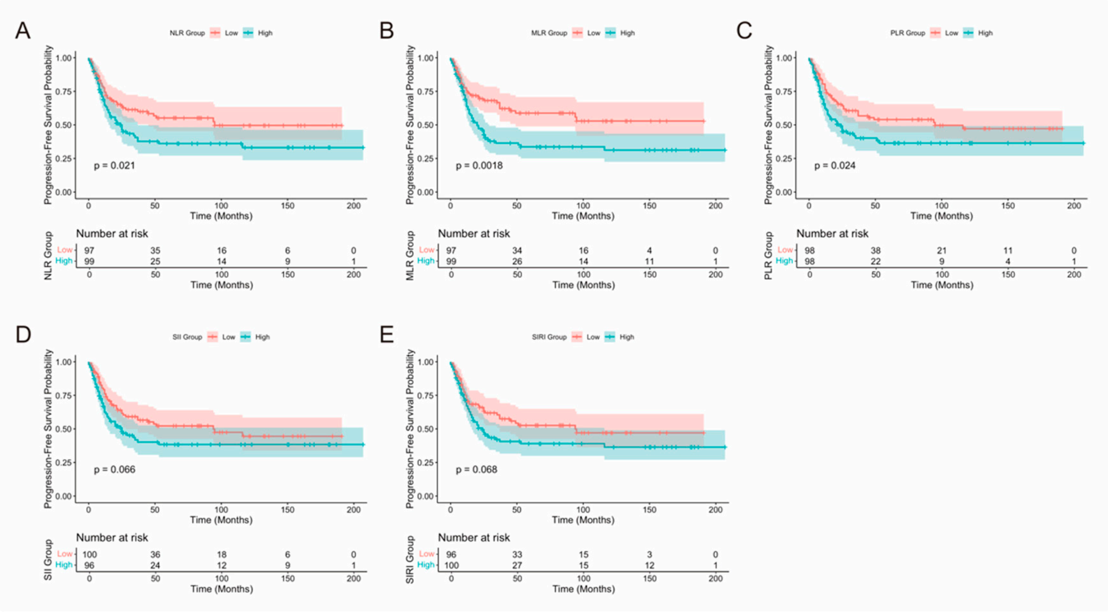

Figure S3 Kaplan–Meier survival curves for PFS in SCNEC patients, showing the prognostic impact of NLR (A), MLR (B), PLR (C), SII (D), and SIRI (E).

PFS, progression-free survival; SCNEC, small cell neuroendocrine carcinoma of the cervix; NLR, neutrophil-to-lymphocyte ratio; MLR, monocyte-to-lymphocyte ratio; PLR, platelet-to-lymphocyte ratio; SII, systemic immune-inflammation index; SIRI, systemic-inflammatory-response index.

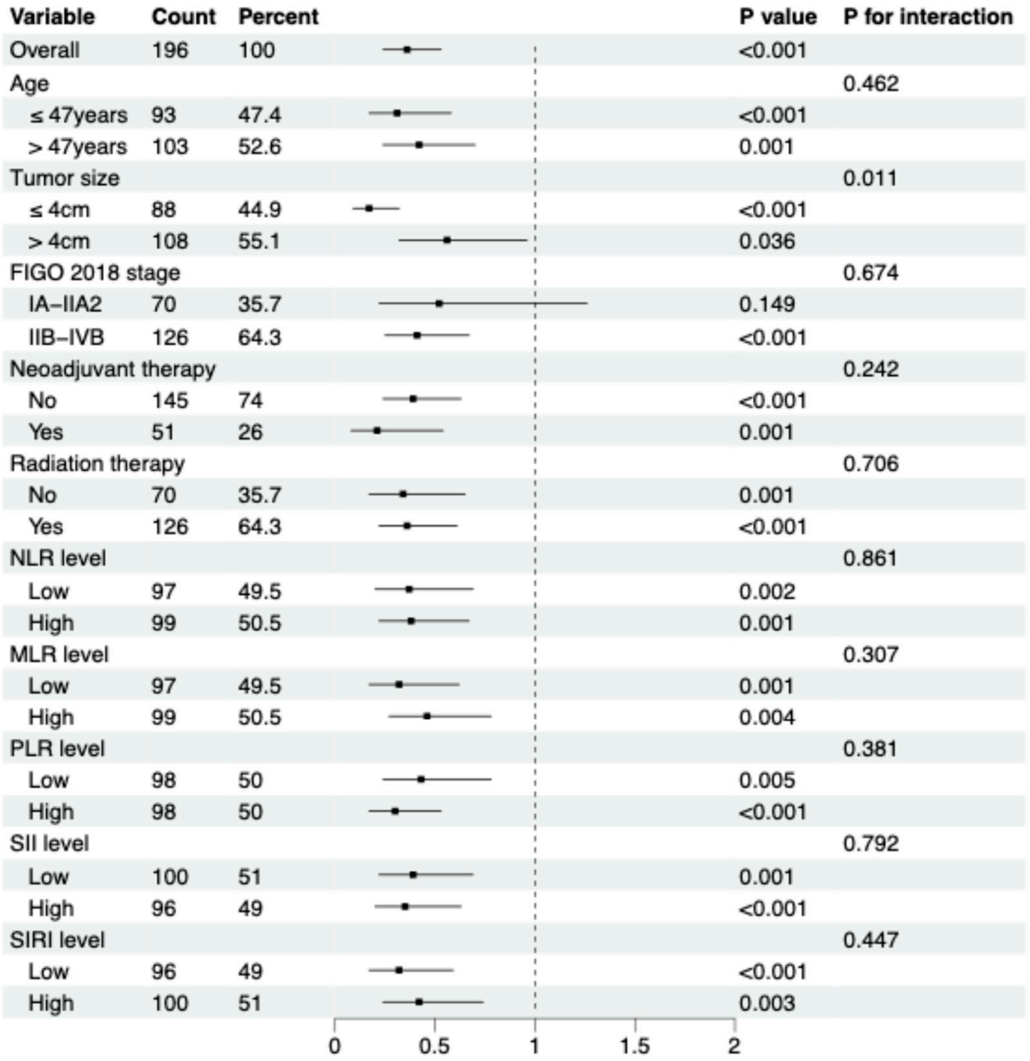

Figure S4 Subgroup analysis of the association between surgical intervention and PFS in SCNEC patients.

PFS, progression-free survival; SCNEC, small cell neuroendocrine carcinoma of the cervix; FIGO, International Federation of Gynecology and Obstetrics; NLR, neutrophil-to-lymphocyte ratio; MLR, monocyte-to-lymphocyte ratio; PLR, platelet-to-lymphocyte ratio; SII, systemic immune-inflammation index; SIRI, systemic-inflammatory-response index.
